# MDT perspective: innovative applications of stereotactic body radiation therapy in metastatic castration-resistant prostate cancer

**DOI:** 10.1038/s41391-024-00922-z

**Published:** 2024-11-18

**Authors:** Andrew W. Hahn, Ana Aparicio, Hossein Jadvar, Darren M. C. Poon

**Affiliations:** 1 Department of Genitourinary Medical Oncology, The University of Texas MD Anderson Cancer Center, Houston, TX USA; 2 Division of Nuclear Medicine and Molecular Imaging Center, Department of Radiology, Keck School of Medicine, University of Southern California, Los Angeles, CA USA; 3 Department of Clinical Oncology, State Key Laboratory of Translational Oncology, Sir YK Pao Centre for Cancer, Hong Kong Cancer Institute, The Chinese University of Hong Kong; Comprehensive Oncology Centre, Hong Kong Sanatorium & Hospital, Hong Kong, China

**Keywords:** Prostate cancer, Cancer therapy

## Summary

The systematic review by Guevelou et al. examines the impact of stereotactic body radiotherapy (SBRT) on patients with metastatic castration-resistant prostate cancer (mCRPC) [1]. The authors analyze 19 relevant studies, predominantly retrospective, to assess the effectiveness of SBRT in improving progression-free survival (PFS) and biochemical progression-free survival (bPFS) in individuals experiencing metachronous oligoprogression, particularly when combined with androgen receptor pathway inhibitors. They report promising outcomes, with bPFS ranging from 9.5 to 17.9 months. While SBRT is noted for its tolerability and low incidence of severe toxicity, the authors emphasize that the overall evidence remains limited, highlighting the necessity for prospective trials to establish clearer clinical guidelines.

## Medical Oncologist’s perspective (Andrew W. Hahn, Ana Aparicio)

For decades, metastasis-directed therapy (MDT) was used solely for palliation in prostate cancer patients. Lately, however, several studies have examined its utility as a systemic therapy-sparing approach and its ability to prevent morbidity and improve outcomes. The ORIOLE, STOMP and EXTEND trials provided evidence that stereotactic body radiotherapy (SBRT), alone or added to intermittent hormone therapy, could prolong overall and eugonadal progression free survival (PFS) in patients with oligometastatic castration sensitive prostate cancers (CSPC) [2–4]. Additionally, the ARTO trial found that adding SBRT to first line abiraterone increased biochemical responses and PFS in patients with oligometastatic CRPC [5]. In patients with various malignancies (including prostate cancer), a randomized phase II trial showed that prophylactic radiation therapy of high-risk asymptomatic bone metastases reduced skeletal related events and hospitalizations and improved overall survival [6]. Thus, given its apparent benefit and low risk in the short-term MDT has been widely adopted (particularly in oligometastatic CSPC), although its long-term safety, effects on survival, and optimal recipients remain to be confirmed.

More recently, the term oligoprogression has come into use to identify targets for MDT. Oligoprogression in CRPC has been further categorized as metachronous (oligometastatic disease in a patient without any prior history of radiographically detectable metastases) or induced (perceived progression in a limited number of metastatic sites in patients with prior history of polymetastatic disease) [1]. However, conceptually, de novo oligometastatic disease in mCSPC, metachronous and induced oligoprogression in mCRPC can all be considered separate points across a spectrum representing resistant clones that may be driving disease progression, the purported target of MDT. The challenge is that the definition of oligoprogression is a moving mark because it is highly dependent on the sensitivity of imaging techniques in use and the number of lesions that can be treated safely. Moreover, although intuitively newly detected lesions should be the ones harboring said resistant clones, this has yet to be rigorously proven. And it is conceivable that the mechanism of benefit from MDT goes beyond eradication of resistant clones (e.g. immune activation) which would make a rigorous definition of oligoprogression less critical and suggest that it is better paired with some systemic therapies over others. Robust prospective trials using a consensus definition of oligoprogression, clinically meaningful endpoints and incorporating correlative studies to interrogate the underlying mechanism of benefit are needed to fully realize the potential of MDT in metastatic cancers.

## Nuclear Radiologist’s perspective (Hossein Jadvar)

Hellman and Weichselbaum proposed a clinical state with limited metastatic burden (oligometastatic disease, or OMD) as an intermediate step in cancer progression from a localized process to a disseminated state [7]. However, a validated molecular signature for OMD remains unspecified. The current identification and characterization of OMD is based on limited (typically up to 5) metastases detected on imaging [8]. Such representation is thus dependent on the sensitivity of the imaging modality [9]. Despite this “moving target” constraint, MDT of OMD, typically performed with SBRT, with or without additional systemic therapy to eradicate occult micrometastases, is viewed as an opportunity for altering the disease trajectory, which may be potentially curative, or delay start of (new) systemic therapy. This notion is based on the presumption that elimination of OMD hinders the ability of cancer to evolve biologically into a more aggressive phenotype and potentially seed other sites [10].

In the clinical domain of prostate cancer, OMD as an umbrella term, has been classified as de-novo (synchronous, coincident with untreated primary tumor at the time of initial staging, and metachronous at the time of biochemical recurrence – oligorecurrent - after definitive primary tumor treatment), and induced (after systemic therapy of polymetastatic disease with few remaining drug-resistant lesions located in pharmacologically privileged sites (oligopersistence), or after an initial favorable response to systemic therapy and subsequent development of disease progression at a limited number of new sites (oligoprogression) [11]. It is recognized that these subclasses of OMD are biologically distinct which may require specific therapeutic approaches [12, 13]. Further, the PRECISE-MDT demonstrated that diverse imaging methods may influence long-term outcomes in OMD treated with MDT [14]. Patients who were treated with prostate-specific membrane antigen positron emission tomography/computed tomography (PSMA PET/CT)-guided MDT had longer PFS compared to those treated with less sensitive choline PET/CT guidance (and by inference guidance by CT and bone scan) [15, 16]. Therefore, in the absence of molecular biomarkers, PSMA PET/CT has been recommended as the imaging modality for clinical decisions relevant to MDT of OMD in prostate cancer [17]. PSMA PET/CT is also appropriate for the imaging assessment of local response to MDT and for restaging [18]. In the relatively near future, there may be additional PET radiotracers based on gastrin-releasing peptide receptor and fibroblast activation protein which may provide synergism to PSMA PET or have utility in those prostate cancers that express no or low PSMA expression [19]. Multi-targeted imaging may offer opportunities for more refined characterization and target-specific MDT in the hopes of attaining favorable patient outcome at low toxicity. Radiopharmaceutical therapy (e.g., 177Lu-PSMA-617) may also be combined with MDT for additional benefit given the encouraging early results of such internal and external radiotherapeutic combination approach [20, 21].

In summary, despite ongoing clinical interest and few randomized trials, there is still more basic research that needs to be done to learn about the underlying biology of the various forms of the OMD clinical state, predictive and prognostic imaging and non-imaging biomarkers, and evidence-based treatment strategies through properly designed and executed clinical trials in order to establish OMD decisively as a clinically distinct and actionable disease state in the management of patients with prostate cancer.

## Radiation Oncologist’s perspective (Darren M.C. Poon)

Historically, radiotherapy was predominantly palliative, e.g. bone pain alleviation, in the management of mCRPC. Recently, a growing body of evidence has supported the incorporation of radical radiotherapy, i.e. SBRT, into the treatment algorithm for mCRPC patients with OMD. Multiple retrospective series have demonstrated that eradication of OMD using SBRT could prevent progression to more aggressive phenotypes and potential tumor seeding to other sites, ultimately enhancing overall disease control [1]. The role of SBRT is further consolidated in the recent phase II ARTO study [5], which showed that combination of SBRT and abiraterone improved biochemical response and progression-free survival in mCRPC patients with OMD compared to abiraterone only. SBRT could also potentially improve overall survival of mCRPC patients with oligoprogressive disease by ablating resistant clones and thus maintaining best responses of other sensitive sites to existing anticancer drugs and delaying the need for next line systemic therapy [1].

Several limitations, however, should be noted for routine clinical use of SBRT in mCRPC patients with OMD. First and foremost, the optimal definition of OMD remains undetermined. Currently, OMD is classified largely based on the number of imaging-identified metastases. The sensitivity of the imaging modality chosen will substantially affect a patient’s OMD status and thus the accuracy and treatment outcome of SBRT. For example, patients with OMD per conventional imaging may be undertreated with SBRT if more lesions are detected on PSMA PET. Another controversy surrounds the appropriate sequence of next-generation imaging, e.g. PSMA-only vs. PSMA–fluorodeoxyglucose (FDG) PET, for screening OMD. Because FDG-positive lesions may be associated with more aggressive phenotypes, the clinical value of dual-tracer PET in patient selection for SBRT warrants further investigation [22]. Lastly, the underlying tumor biology of OMD remains elusive. Genomic biomarkers of mCRPC that help to predict response to SBRT or inherent resistance to radiation should be further studied.

With the continuous advancement of radiation techniques, including motion management, image guidance, and online adaptive planning, SBRT precision keeps improving. As a non-interventional metastasis-directed therapy, SBRT is a generally safe treatment approach for OMD, with a <5% incidence of grade ≥3 complications [1]. Despite the rapid expansion of systemic therapies for mCRPC, patients might inevitably exhaust all available regimens because of treatment resistance. Recent evidence has shown that SBRT may extend the time to switch to next line systemic therapy, potentially improving patient survival and financial burden. The SABR-COMET study [23] demonstrated that SBRT was more cost-effective than standard of care in treating OMD. Economically, the limited fractionation and expenses of SBRT are highly attractive compared with costly novel therapies.

Multiple prospective studies are evaluating the role of SBRT in mCRPC patients receiving androgen receptor pathway inhibitors [1]. Future trials are warranted to investigate the combination of SBRT and poly (ADP-ribose) polymerase inhibitors or radioligand therapy, which are increasingly available novel therapies for mCRPC. As SBRT is promising and safe, the role of radiation oncologists in the multidisciplinary management of mCRPC should become more radical rather than palliative.
